# Diabetic polyneuropathy, sensory neurons, nuclear structure and spliceosome alterations: a role for CWC22

**DOI:** 10.1242/dmm.028225

**Published:** 2017-03-01

**Authors:** Masaki Kobayashi, Ambika Chandrasekhar, Chu Cheng, Jose A. Martinez, Hilarie Ng, Cristiane de la Hoz, Douglas W. Zochodne

**Affiliations:** 1 Division of Neurology and Department of Medicine, Faculty of Medicine and Dentistry, and the Neuroscience and Mental Health Institute, University of Alberta, Edmonton, Canada, T6G 2G3; 2 Hotchkiss Brain Institute andDepartment of Clinical Neurosciences, Faculty of Medicine, University of Calgary, Canada, T2N 4N1

**Keywords:** Diabetic polyneuropathy, Sensory ganglia, SMN complex proteins, Gemini of coiled bodies, Small nuclear ribonucleoproteins

## Abstract

Unique deficits in the function of adult sensory neurons as part of their early neurodegeneration might account for progressive polyneuropathy during chronic diabetes mellitus. Here, we provide structural and functional evidence for aberrant pre-mRNA splicing in a chronic type 1 model of experimental diabetic polyneuropathy (DPN). Cajal bodies (CBs), unique nuclear substructures involved in RNA splicing, increased in number in diabetic sensory neurons, but their expected colocalization with survival motor neuron (SMN) proteins was reduced – a mislocalization described in motor neurons of spinal muscular atrophy. Small nuclear ribonucleoprotein particles (snRNPs), also participants in the spliceosome, had abnormal multiple nuclear foci unassociated with CBs, and their associated snRNAs were reduced. CWC22, a key spliceosome protein, was aberrantly upregulated in diabetic dorsal root ganglia (DRG), and impaired neuronal function. CWC22 attenuated sensory neuron plasticity, with knockdown *in vitro* enhancing their neurite outgrowth. Further, axonal delivery of CWC22 siRNA unilaterally to locally knock down the aberrant protein in diabetic nerves improved aspects of sensory function in diabetic mice. Collectively, our findings identify subtle but significant alterations in spliceosome structure and function, including dysregulated CBs and CWC22 overexpression, in diabetic sensory neurons that offer new ideas regarding diabetic sensory neurodegeneration in polyneuropathy.

## Abstract (precis)

**Summary:** Subtle alterations in spliceosome structure and function in diabetic sensory neurons, including dysregulated Cajal bodies and CWC22 overexpression, might promote the development of diabetic sensory polyneuropathy.

## INTRODUCTION

Distal, symmetric sensory polyneuropathy (DPN) is the most common form of diabetic neuropathy, associated with numbness and neuropathic pain that starts in distal nerve territories (Brown and Asbury, 1984; Zochodne, 2007). Dorsal root ganglion (DRG) sensory neurons are targeted by diabetes but can be supported through direct neuronal insulin signalling of sensory neurons, GLP-1 agonism, acting as a neuron growth factor, or HSP27 overexpression. Knockdown of PTEN overexpression in diabetic sensory neurons can compensate for their regenerative failure (Brussee et al., 2004; Kan et al., 2012; Korngut et al., 2012; Singh et al., 2014; Xu et al., 2004; Zochodne, 2015).

The role of altered nuclear structure and function has received lesser attention in sensory neuron degeneration in diabetes. Within the nuclear interchromatin space, unique membraneless subnuclear organelles – referred to as nuclear bodies such as nucleoli, Cajal bodies (CBs), and nuclear speckles – spatially compartmentalize the microenvironment to facilitate more efficient changes of gene expression (Mao et al., 2011). Nucleoli act as a central hub for coordinating the response to stress (Boulon et al., 2010). CBs participate in crosstalk with nucleoli upon cellular stress and are prominent in proliferative and metabolically active cells such as cancer cells or neurons (Machyna et al., 2013; Palanca et al., 2014; Strzelecka et al., 2010a). CBs concentrate small nuclear ribonucleoprotein particles (snRNPs), key components of spliceosome, and increase the efficiency of gene expression through pre-mRNA splicing (Klingauf et al., 2006; Strzelecka et al., 2010b). Nuclear speckles accumulate snRNPs and other non-snRNP protein-splicing factors, and provide a place to execute splicing (Girard et al., 2012). The overall role of these nuclear bodies in subtle diabetic neurodegeneration, however, is unexplored (Morimoto and Boerkoel, 2013).

Survival motor neuron protein (SMN), localized in nuclear foci, functions in the assembly of snRNPs in collaboration with CBs (Hebert et al., 2001; Liu and Dreyfuss, 1996; Matera and Wang, 2014; Narayanan et al., 2004). SMN mutations underlie spinal muscular atrophy (SMA) through defects in motor neuron CB formation, the assembly of snRNPs and pre-mRNA splicing defects (Girard et al., 2006; Lunn and Wang, 2008; Zhang et al., 2008). However, SMN-deficient sensory neurons *in vitro* are also abnormal with shorter neurites and small growth cones (Jablonka et al., 2006).

In the present study, we identify evidence for unique alterations in nuclear structure and function, particularly in the spliceosome, that accompany diabetic sensory neurodegeneration: rises in CBs, declines in CB SMN proteins, abnormal distribution and expression of snRNPs and snRNAs, and aberrant upregulation of CWC22, a spliceosomal exon junction complex (EJC) assembly protein (Barbosa et al., 2012; Steckelberg et al., 2012). Knockdown of CWC22 altered the growth properties of sensory neurons and the phenotype of experimental DPN. Our findings provide evidence that spliceosome dysregulation might be a key neurodegenerative feature of DPN.

## RESULTS

### Diabetes impairs nerve conduction velocity and causes atrophy of DRG sensory neurons and their nuclei

**Fig. 1.**
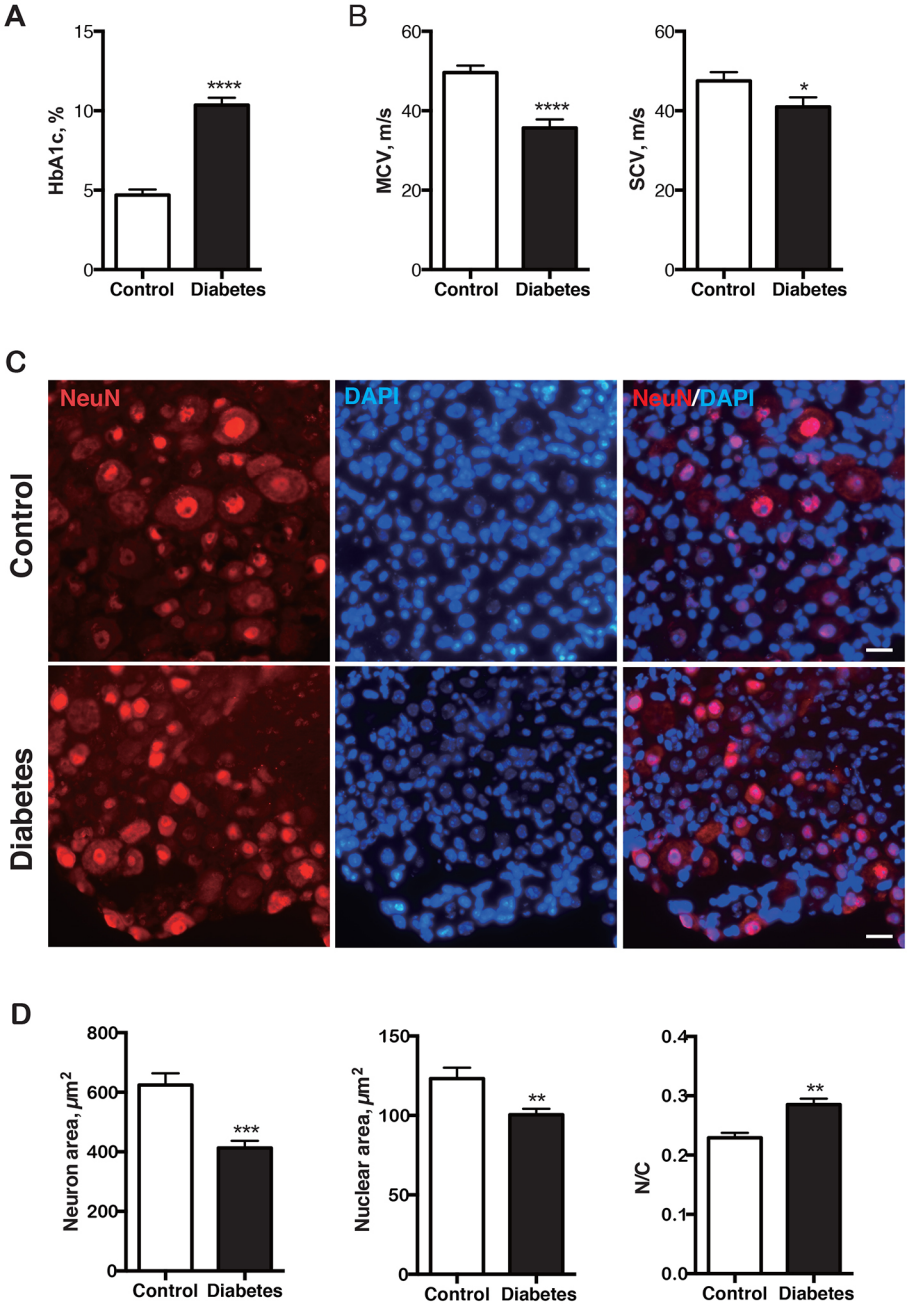
**Nerve conduction slowing and DRG sensory neuron and nuclear atrophy in 16-week-old diabetic mice.** (A) HbA1c was increased in diabetic mice (*n*=16) compared with nondiabetic controls (*n*=13). (B) Motor and sensory conduction velocities of diabetic mice were decreased compared with controls. Motor nerve: control, *n*=13; diabetes, *n*=11. Sensory nerve: control, *n*=13; diabetes, *n*=7. (C) Representative images of DRG sensory neurons stained with anti-NeuN antibody and DAPI in diabetic mice and controls. Scale bar: 20 μm. Diabetic sensory neurons were atrophic. (D) The average size of neuron and nucleus, and the nuclear-to-cytoplasmic (N/C) ratio of DRG neurons in diabetic mice (*n*=5) and controls (*n*=5). Both neuronal and nuclear size were smaller in diabetic mice than in controls, whereas N/C ratio was larger in diabetic mice. *P<0.05, **P<0.01, ***P<0.001, ****P<0.0001, unpaired one-tailed Student's *t*-test. Data represented as mean±s.e.m.

Diabetic mice at 16 weeks after induction had hyperglycemia, elevated HbA1c levels (Fig. 1A) and reductions of motor and sensory nerve conduction velocity (Fig. 1B). In DRG sensory neurons, both neuronal and nuclear size were smaller in diabetic mice, whereas the nuclear-to-cytoplasmic ratio was significantly larger (Fig. 1C,D). Nuclear atrophy, accompanying that of the perikarya generally, indicates an overall alteration in its structure that might relate to function (Brussee et al., 2004; Kan et al., 2012; Kishi et al., 2002).

### Diabetes increases CB number in the nuclei of DRG sensory neurons

**Fig. 2.**
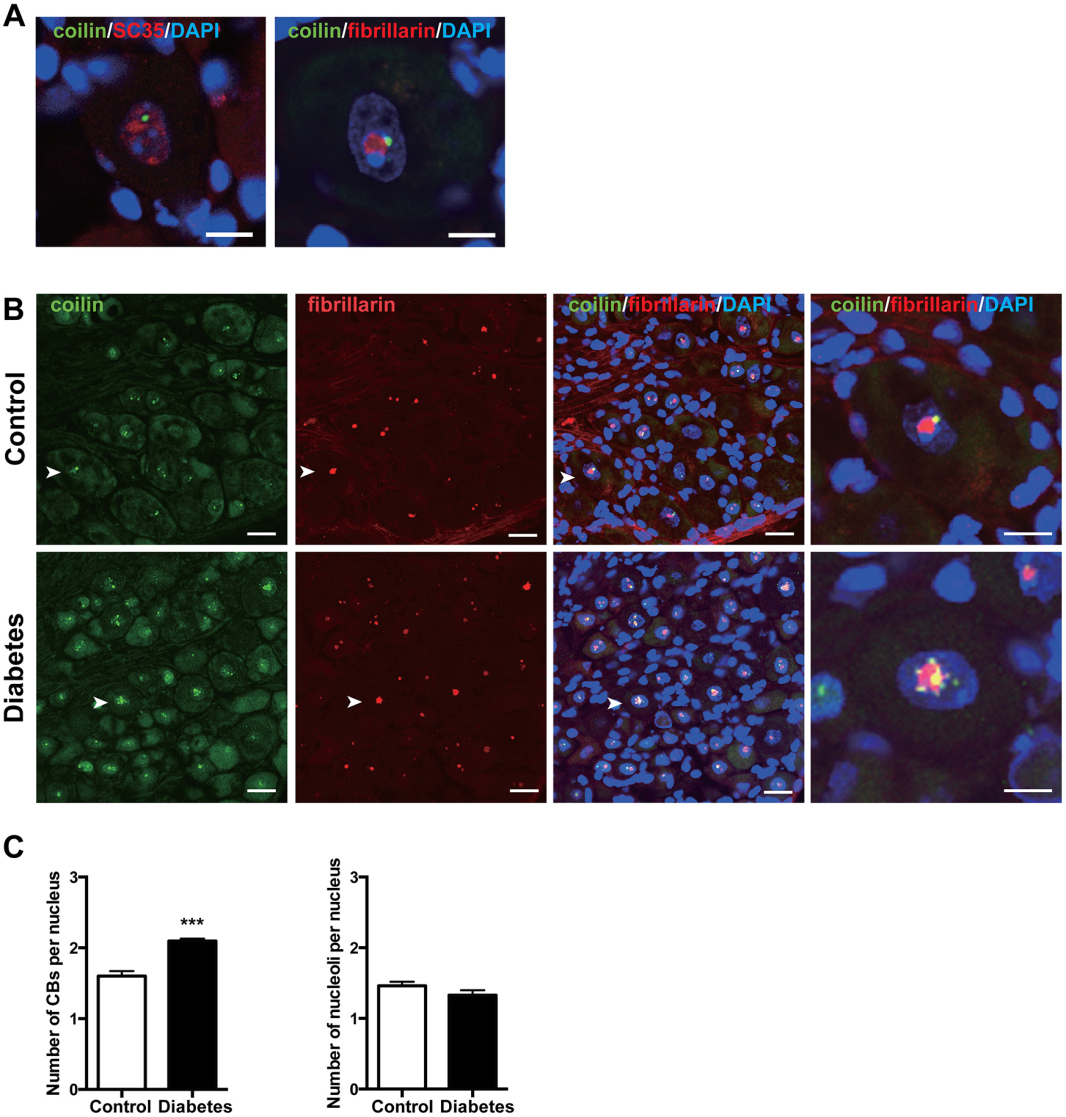
**CB number is increased in diabetic sensory neurons.** (A) Normal distribution of CBs (coilin), nucleoli (fibrillarin) and nuclear speckles (SC35) in DRG sensory neurons. A single CB was in contact with a nucleolus and nuclear speckles were located in the interchromatin regions of the nucleoplasm in controls. Scale bar: 10 μm. (B) Normal and pathological distribution of CBs and nucleoli in sensory neurons. A number of CBs were localized around a nucleolus throughout the nucleoplasm in diabetic nuclei. Arrowheads indicate sensory neurons magnified in the insets. Scale bar: 20 μm, 10 μm in magnified panels far right. (C) Number of CBs and nucleoli per nucleus in diabetic mice (*n*=5) and controls (*n*=5). The number of CBs increased in diabetic nuclei, and the number of nucleoli was unchanged in diabetic nuclei. ***P<0.001, unpaired two-tailed Student's *t*-test. Data represented as mean±s.e.m.

In nondiabetic DRG sensory neurons, a single CB expressing coilin was usually adjacent to a fibrillarin-labelled nucleolus. Nuclear speckles, marked by SC35 protein, occupied the interchromatin space of the nucleoplasm (Fig. 2A). In diabetic sensory neurons, numerous CBs were instead distributed adjacent to nucleoli and found throughout the nucleoplasm (Fig. 2B). The number of CBs, but not nucleoli per neuron, was increased in diabetes (Fig. 2C). Nucleoli and nuclear speckles were not structurally altered.

### Loss of CB colocalization with SMN foci in the nuclei of diabetic DRG sensory neurons

**Fig. 3.**
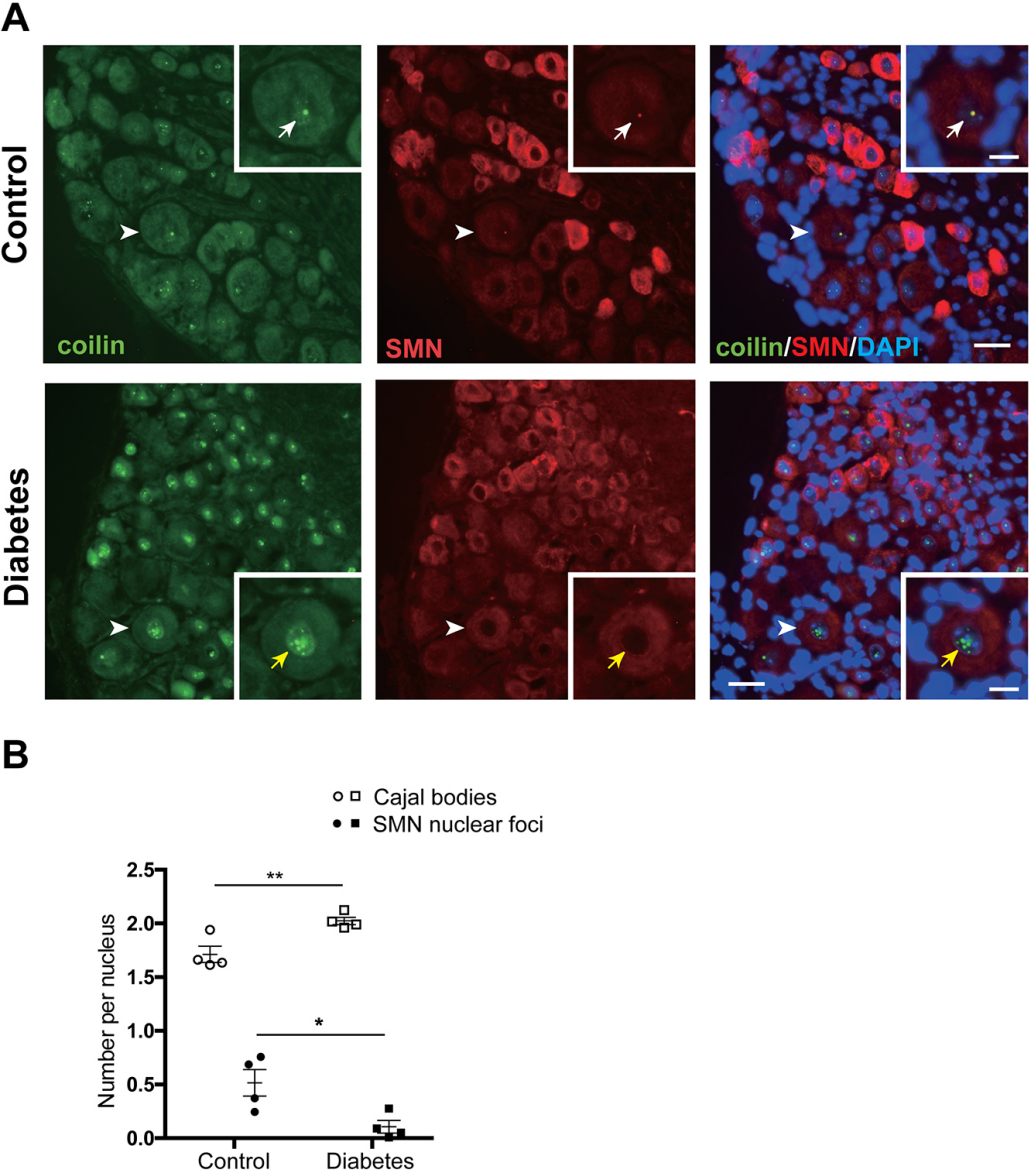
**CBs lose their colocalization with SMN nuclear foci in diabetic mice.** (A) Normal and pathological distribution of SMN proteins in sensory neurons. SMN proteins were found throughout the cytoplasm and as nuclear foci in control sensory neurons. SMN nuclear foci localized within CBs (coilin) in controls (upper panels, white arrows), whereas in the diabetic nucleus the numerous CBs were present but lost their colocalization with SMN nuclear foci (lower panels, yellow arrows). Arrowheads indicate sensory neurons magnified in the insets. Scale bars: 20 μm, 10 μm in insets. (B) Quantitative analysis of colocalization of CBs and SMN nuclear foci in sensory neurons. The number of CBs per nucleus was significantly increased in diabetic mice (*n*=4), whereas the number of SMN foci colocalized with CBs was significantly decreased in diabetic mice (*n*=4), compared with controls (*n*=4). *P<0.05, **P<0.01, unpaired two-tailed Student's *t*-test. Data represented as mean±s.e.m.

We next examined the distribution of CBs and SMN proteins in sensory neurons. In nondiabetic mice SMN proteins were distributed in CB-associated nuclear foci and throughout the cytoplasm as described in motor neurons (Liu and Dreyfuss, 1996) (Fig. 3A, upper panels). In diabetic mice, numerous CBs lost their colocalization with SMN nuclear foci (Fig. 3A, lower panels). Whereas total numbers of CBs per nucleus increased in diabetic mice, the number of CBs containing SMN declined by ∼80% (Fig. 3B), suggesting loss of recruitment of SMN. The loss of nuclear SMN is a key finding in motor neurons of SMA (Lefebvre et al., 1997).

### Alterations of snRNPs and snRNAs in diabetic neurons

**Fig. 4.**
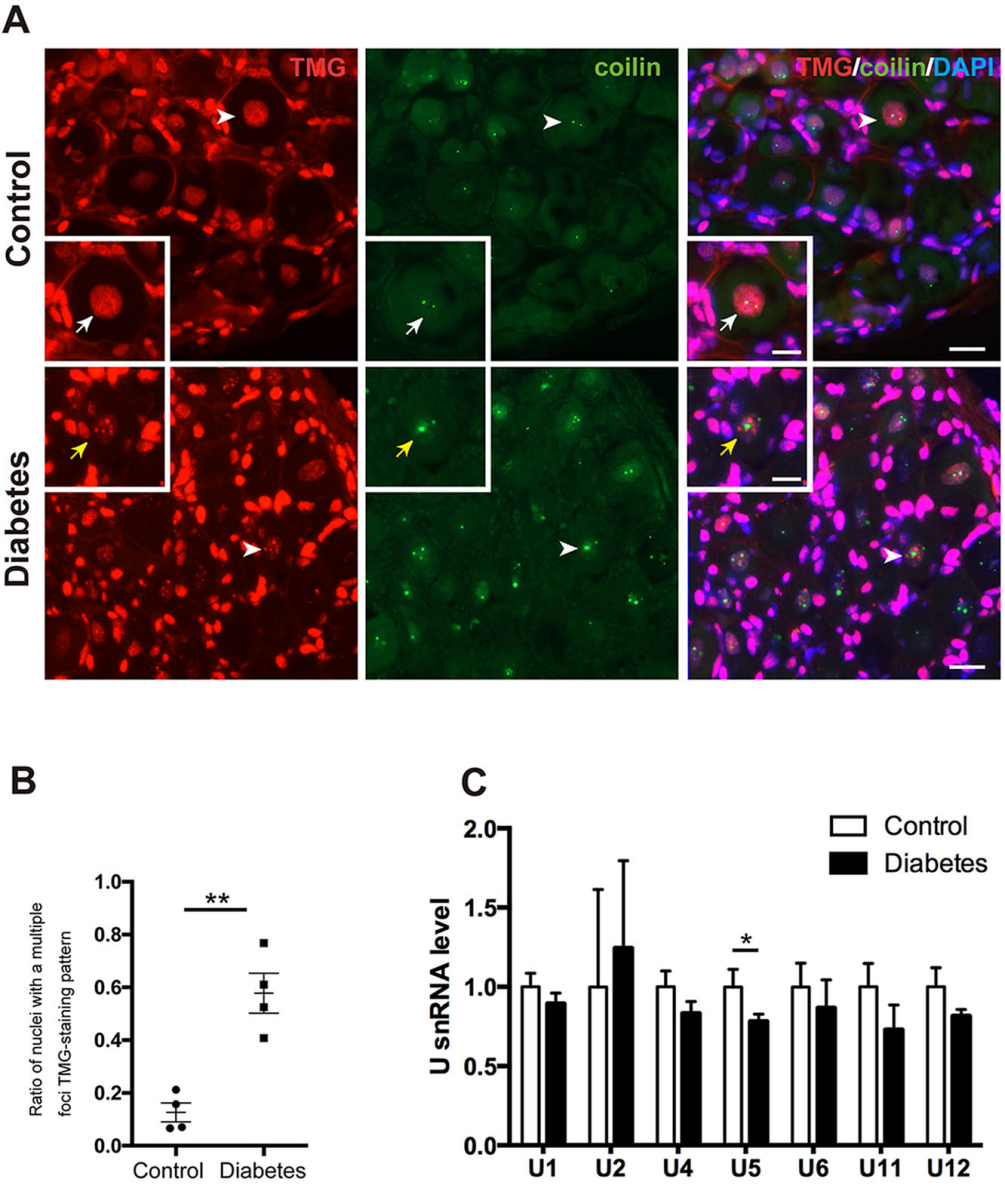
**snRNPs form multiple foci with reduction of U5 snRNA and lose their colocalization with CBs in the nuclei of diabetic sensory neurons.** (A) TMG staining patterns and relation to CBs (coilin) in diabetic and control mice. snRNPs immunostained for TMG were present homogeneously throughout the nucleoplasm and colocalized with CBs in controls (white arrows), whereas in the diabetic nuclei snRNPs formed multiple foci that did not colocalize with CBs (yellow arrows). Arrowheads indicate sensory neurons magnified in the inset. Scale bar: 20 μm, 10 μm in insets. (B) The ratio of nuclei with snRNPs forming multiple foci was increased in diabetes (*n*=4) compared with controls (*n*=4). (C) U snRNA levels in normal (*n*=6) and diabetic DRGs (*n*=6). There is a trend towards reduced snRNAs in diabetic sensory neurons. The U5 snRNA level was significantly reduced in diabetic mice compared with controls. *P<0.05, **P<0.01, unpaired one-tailed Student's *t*-test. Data represented as mean±s.e.m.

We then asked whether diabetes compromises snRNP biogenesis. snRNPs were labelled by TMG, a modified nucleoside at the 5′-cap site of snRNAs (Matera and Wang, 2014; Strzelecka et al., 2010b). In nondiabetic DRG sensory neurons, snRNPs were homogeneous in the nucleus and colocalized with CBs. In diabetic neurons, snRNPs aggregated into multiple foci and were dissociated with CBs (Fig. 4A) and the ratio of nuclei with abnormal snRNP multiple foci was increased (Fig. 4B). There were trends toward reduced snRNAs of both major (U1, U2, U4, U5 and U6) and minor (U11, U12) classes and a significant decline in U5 snRNA in diabetic sensory neurons. Overall, the findings resembled aberrant snRNP accumulation in motor neuron nuclei of amyotrophic lateral sclerosis (ALS) and loss of snRNPs in SMA (Tsuiji et al., 2013).

### CWC22 is upregulated in diabetic DRG sensory nuclei

**Fig. 5.**
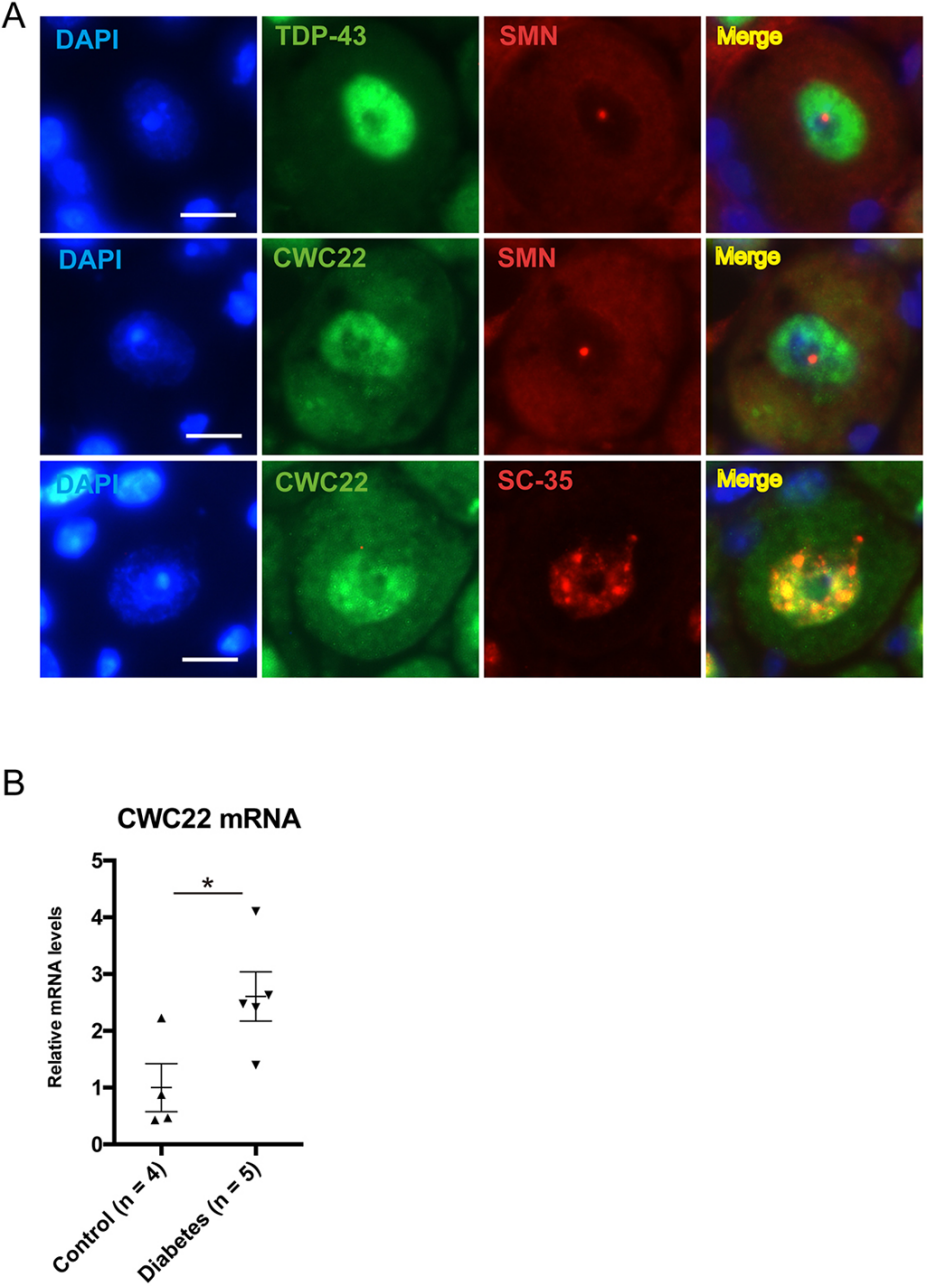
**CWC22 is colocalized with nuclear speckles and upregulated in diabetic DRG sensory neurons.** (A) Subcellular distribution of TDP-43 and CWC22 in control DRG sensory neurons. TDP-43 was stained diffusely in the nucleus, excluding SMN foci in sensory neurons. CWC22 consistently colocalized with a marker protein SC35 of nuclear speckles in sensory neurons. No obvious differences in the subcellular localization of CWC22 were identified in diabetic neurons (not shown) compared with controls. Scale bar: 10 μm. (B) qRT-PCR analysis of *Cwc22* mRNA expression in diabetic and control mice. *Cwc22* expression was upregulated ∼2.5-fold in diabetic DRGs. *P<0.05, unpaired two-tailed Student's *t*-test. Data represented as mean± s.e.m. See Cheng et al. (2015) for microarray data indicating rises in *Cwc22* expression as reported separately.

The subcellular distribution of two key factors related to pre-mRNA splicing, TDP-43 (also known as TARDBP) and CWC22, was examined in sensory neurons (Fig. 5A). TDP-43, a DNA/RNA-binding protein mislocalized in ALS, had a normal diffuse distribution in diabetic sensory neurons (Ling et al., 2013) (Fig. 5A). In a separately reported microarray gene-expression study of DRGs mRNA and miRNA in chronic diabetes, we identified 24 differentially altered mRNAs of 28,669 screened (Cheng et al., 2015). Included among those upregulated in diabetes was *Cwc22*, an mRNA splicing factor located in nuclear speckles (Steckelberg et al., 2012). Here, we confirmed by qRT-PCR *Cwc22* mRNA upregulation of 2.5-fold in diabetic DRGs (Fig. 5B). CWC22 protein expression almost entirely overlapped with that of nuclear speckles, and did not colocalize with SMN. Despite upregulation of its mRNA, its distribution was not apparently altered in diabetic sensory neurons (Fig. 5A).

### CWC knockdown is associated with improved outgrowth in adult sensory neurons

**Fig. 6.**
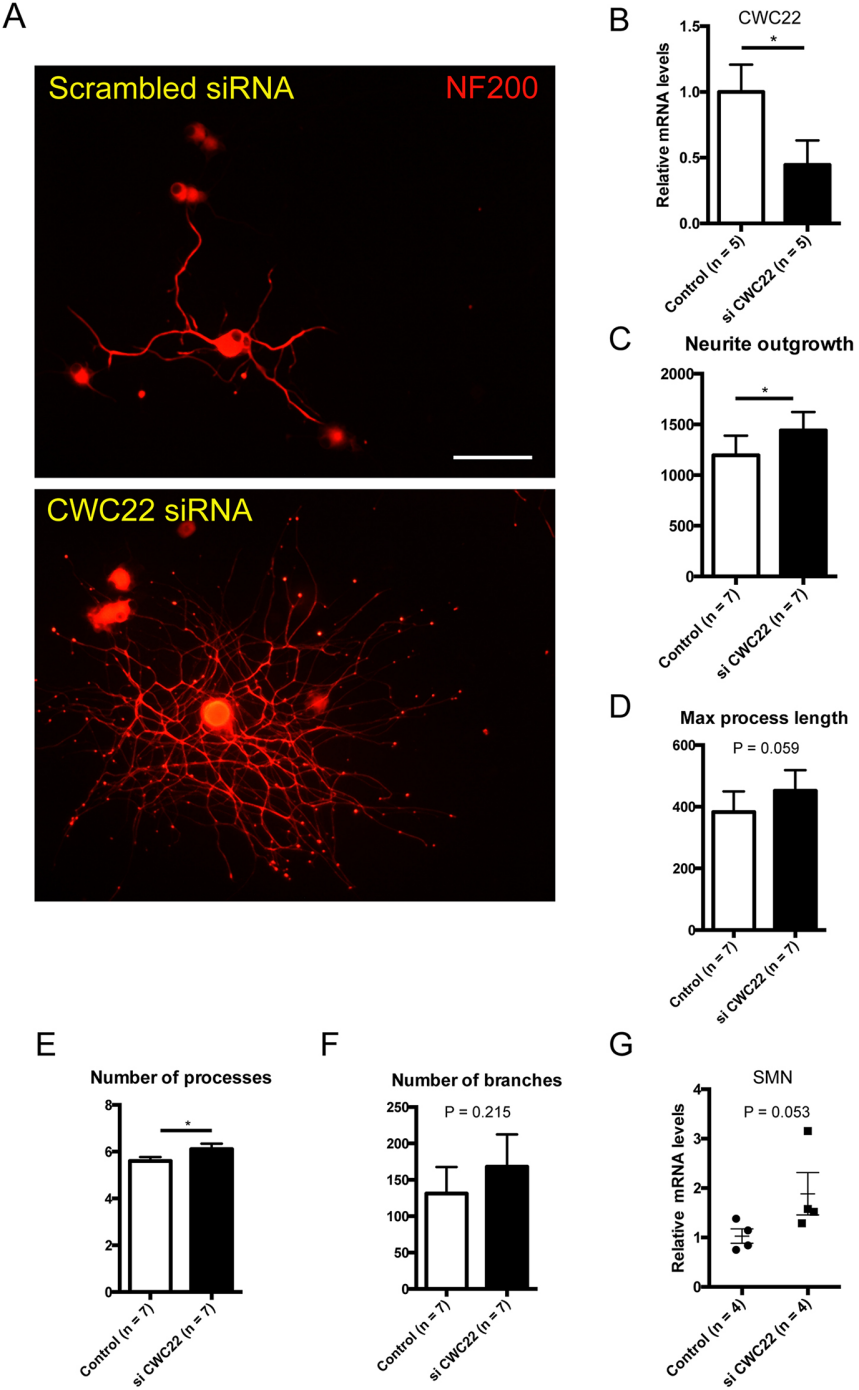
**siRNA knockdown of CWC22 increases DRG neurite outgrowth.** (A) Representative images of DRG neurons with scrambled siRNA or CWC22 siRNA. Note the increase in neurite outgrowth. Scale bar: 100 μm. (B) CWC22 siRNA significantly decreased *Cwc22* mRNA in adult DRG sensory neurons. *P<0.05, unpaired one-tailed Student's *t*-test. (C) Neurite outgrowth (total length of candidate neurites). Scrambled: 1195±193 (mean±s.e.m.), siCWC22: 1440±182; *n*=7, *P=0.044, paired two-tailed Student's *t*-test. (D) Maximum process length (maximal length over all processes). Scrambled: 383±67, siCWC22: 452±67; *n*=7, *P*=0.059, paired two-tailed Student's *t*-test. (E) Number of processes (number of main neurites sprouting from the cell body). Scrambled: 5.60±0.17, siCWC22: 6.11±0.23; *n*=7, *P=0.011, paired two-tailed Student's *t*-test. (F) Number of branches (total branch points). Scrambled: 131±36, siCWC22: 168±44; *n*=4, *P*=0.2, paired two-tailed Student's *t*-test. (G) CWC22 siRNA was associated with a borderline increase in *Smn* mRNA. Scrambled: 1.03±0.14, siCWC22: 1.89±0.43; *n*=4, *P*=0.053, unpaired one-tailed Student's *t*-test.

To gauge what the overall impact might be of CWC22 expression on the growth properties of normal adult sensory neurons, we examined the impact of CWC22 knockdown, using siRNA *in vitro* (Fig. 6A-F). CWC22 siRNA knockdown after transfection was confirmed at the mRNA level (Fig. 6B). CWC22 knockdown was associated with rises in neurite outgrowth and in their numbers of processes (Fig. 6C,E), whereas process length showed a borderline rise and the number of branches remained unchanged (Fig. 6D,F). Additionally, *Smn* mRNA expression was not significantly affected by CWC22 knockdown (Fig. 6G). These findings provide evidence that links aberrant protein expression within the spliceosome to the plasticity properties of neurons.

### Local CWC22 siRNA unilaterally improves features of experimental diabetic polyneuropathy

**Fig. 7.**
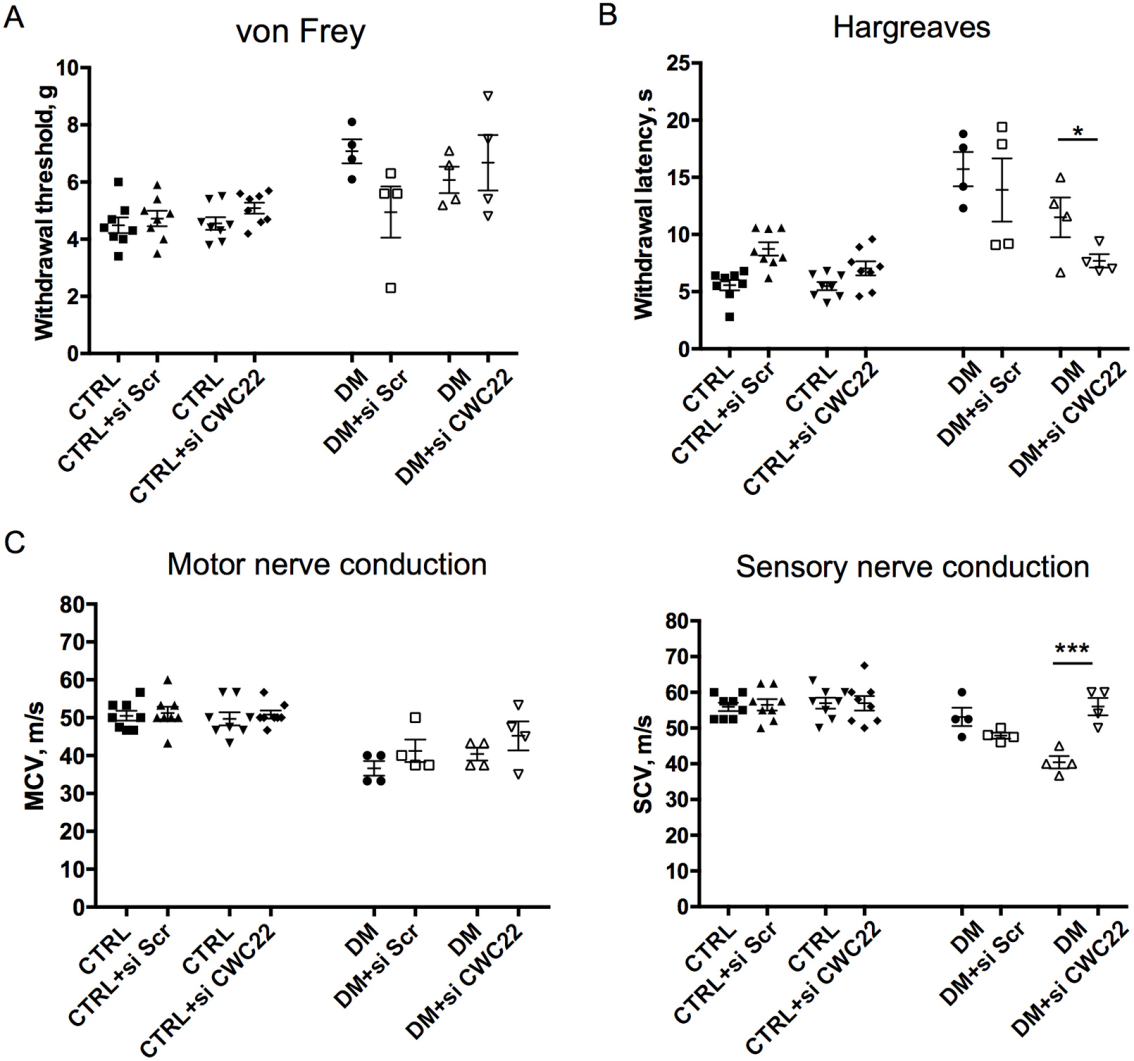
**Local knockdown of CWC22 improves indices of sensory neuron functions in diabetes.** Diabetic mice (DM) and control mice were treated by unilateral near-nerve and intraplantar injection of siRNA to silence CWC22 in one limb and control scrambled siRNA in the contralateral limb. (A) Mechanical sensitivity test; von Frey test. (B) Thermal sensitivity test; Hargreaves test. Thermal sensitivity was improved in the limb exposed to CWC22 siRNA. (C) Motor and sensory conduction studies before and after the injection of CWC22 siRNA or scrambled siRNA. Sensory conduction velocity improved in the limb exposed to CWC22 siRNA. Control+siCWC22, *n*=8; control+siScr, *n*=8; DM+siCWC22, *n*=4; DM+siScr, *n*=4. *P<0.05, ***P<0.001, unpaired one-tailed Student's *t*-test. Data represented as mean±s.e.m.

To examine whether inhibition of CWC22 upregulation in diabetes could alter the diabetic phenotype, we utilized local nonviral siRNA to downregulate its expression unilaterally in the hindlimb of diabetic and nondiabetic mice *in vivo*. Diabetic mice underwent intraplantar and near-nerve siRNA delivery in one limb on days 0, 2, 4 and 6, with contralateral injection of scrambled sequence control siRNA on the same days into the contralateral side. This approach allows close comparison of neurological function between limbs in the same animal, a methodology previously validated (Christie et al., 2014; Guo et al., 2011; Singh et al., 2014; Singhal et al., 1997). Mechanical sensitivity, diminished in diabetic mice, was unaltered by the intervention (Fig. 7A). In contrast, thermal sensitivity, similarly impaired in diabetics, was improved unilaterally in the limb exposed to CWC22 siRNA (Fig. 7B). Slowing of motor conduction velocity secondary to diabetes was not changed following CWC22 siRNA but there was an improvement in ipsilateral sensory conduction velocity (Fig. 7C). Overall, the findings did identify selective improvements by *Cwc22* mRNA knockdown on established abnormalities of a chronic diabetic neuropathy model.

## DISCUSSION

In this study, we have shown that diabetes alters several nuclear proteins required for pre-mRNA splicing in DRG sensory neurons, changes that might contribute to sensory neuron degeneration. Diabetic DRG sensory nuclei undergo rises in CBs, reductions of associated SMN, an abnormal distribution of snRNPs and depleted snRNAs. Inhibition of a key splicing factor, CWC22, that is aberrantly upregulated in diabetes and impacts the growth properties of sensory neurons, improves features of DPN.

Neuronal atrophy in our model was consistent with prior work in rats (Kishi et al., 2002). Perikaryal and nuclear caliber, reflecting an overall appraisal of their status in diabetes, might not translate into functional significance but does indicate that diabetes targets these components of the neuronal tree. These changes do not always evolve into overt neuron loss (Cheng and Zochodne, 2003), but include a series of molecular changes related to structure, neuronal stress and protection (Zochodne, 2015). Neuron loss up to ∼30% is observed in longer models, up to 9 months of diabetes, but we believe the earlier timepoint in the present work reflects active neurodegeneration rather than changes in residual neurons.

Loss of CBs results in splicing defects and embryonic lethality in zebrafish (Strzelecka et al., 2010b). CBs are required for snRNP assembly and are thought to increase the efficiency of gene expression by facilitating splicing (Andrade et al., 1991; Berciano et al., 2007; Boulon et al., 2010; Klingauf et al., 2006; Palanca et al., 2014; Strzelecka et al., 2010a). It is plausible that an increase of CB number might reflect the increased mRNA demand in sensory neurons under diabetic ‘stress’.

SMN proteins concentrated in nuclear foci are frequently adjacent to or overlap CBs, indicating a close functional relationship. SMN CBs are known as Gemini of CBs or ‘gems’ (Cauchi, 2010) and they decline in SMA motor neurons (Grzeschik et al., 2005; Lefebvre et al., 1997; Lunn and Wang, 2008; Young et al., 2000). In ALS mice with mutant SOD1, the subcellular localization of SMN proteins is altered, preventing its recruitment to CBs and gems (Kariya et al., 2012). We identified declines in gems in diabetic sensory neurons, resembling the change seen in ALS, which might contribute to sensory neurodegeneration. Importantly, sensory neurons are also known to be targets, as studied in models, isolated sensory neurons and in patients with SMA (Jablonka et al., 2006; Rudnik-Schöneborn et al., 2003).

snRNPs formed abnormal multiple foci in diabetic nuclei, had impaired colocalization with CBs and there were declines in U5 snRNA. In normal cells, snRNPs assembled with SMN proteins in cytosol are imported into the nucleus, and their final assembly is promoted in CBs (Matera and Wang, 2014). U4/U6.U5 tri-snRNPs accumulate in CBs, and inhibition of tri-snRNPs assembly triggers CB formation (Novotný et al., 2015). Given these findings, our reduction of U5 snRNA in diabetic sensory neurons might explain the concurrent rise of CB number we observed. Colocalization of snRNPs and CBs promotes pre-mRNA splicing, and SMN deficiency induces loss of snRNPs in SMA (Zhang et al., 2008). Moreover, snRNPs aberrantly accumulate in motor neuron nuclei in ALS (Tsuiji et al., 2013). Therefore, the aggregation of snRNPs and reduction of U5 snRNAs associated with loss of gems support misregulation of pre-mRNA splicing in diabetic sensory neurons.

We anticipated a range of additional protein participants in diabetic sensory neuron spliceosomes that have altered function. In keeping with this, our DRG mRNA array did identify aberrant CWC22, an essential splicing factor in diabetes. Although CWC22-depleted cells show impaired pre-mRNA splicing (Steckelberg et al., 2012) and downregulation of diverse gene expression pathways (Steckelberg et al., 2015), the putative role of chronic overexpression is unknown. We found that knockdown of *Cwc22* mRNA enhanced outgrowth in dissociated adult DRG sensory neurons, and improved selected indices of sensory nerve function in diabetic mice. Therefore, it is possible that the biological role of CWC22 in sensory neurons might, in normal circumstances, attenuate neuron plasticity through downregulation of a range of growth-related genes. Identifying further connections between this single misregulated protein and the neuronal phenotype, both normal and diabetic, will be important.

Collectively, our findings support the concept that diabetes directly targets sensory neurons, particularly nuclear structure and function, in rendering neuropathy. The increases of CWC22 and CBs expression we observed support the concept that a significant part of this dysfunction involves aberrant pre-mRNA splicing demand. Loss of SMN and snRNPs colocalized with CBs might cause misregulation of pre-mRNA splicing leading to sensory neuron degeneration, not unlike the more overt role of this pathway in the motor neurons of SMA or ALS. Taken together, our findings identify novel degenerative mechanisms that include new molecular targets relevant to diabetic neuropathy.

## MATERIALS AND METHODS

### Induction of diabetes

All protocols were reviewed and approved by the University of Calgary Health Sciences Animal Care Committee following guidelines by the Canadian Council of Animal Care. C57B6/L mice (4 weeks of age, 19 to 21 g) were used in this study. Diabetes was induced by intraperitoneal injections of 85, 70 and 55 mg/kg streptozotocin (STZ) (Sigma) dissolved in citrate buffer (pH 4.5) for three consecutive days as previously described (Cheng et al., 2015). Control mice were injected with citrate buffer. Diabetes was defined as fasting glucose level of 16 mmol/l.

### Electrophysiology, behavioural testing

Multifibre motor and sensory nerve conduction recordings were carried out in sciatic–tibial nerves in mice anaesthetized with isoflurane at a near-nerve subcutaneous temperature of 37°C as previously described (Kan et al., 2012). Thermal and mechanical sensitivity were measured as previously described (Kan et al., 2012).

### Immunohistochemistry

Harvested DRGs (L4 and L5) were placed in modified Zamboni's solution overnight at 4°C. The samples were then rinsed in PBS three times and suspended in PBS–20% sucrose solution overnight at 4°C. After embedding in OCT compound, 10-μm thick sections were placed onto poly-L-lysine-coated glass slides. The primary antibodies applied were: mouse anti-NeuN (1:100; MAB377, Millipore) for detection of neuronal nuclei, mouse anti-fibrillarin (1:100; sc-374022, Santa Cruz Biotechnology) for nucleoli, rabbit anti-coilin (1:100; sc-32860, Santa Cruz Biotechnology) for CBs, mouse anti-SMN (1:200; NB100-1936, Novus Biologicals), mouse anti-SC35 (1:500; ab11826, Abcam) for nuclear speckles, rabbit anti-CWC22 (1:100; HPA036748, Sigma-Aldrich), rabbit-anti-TARDBP antibody, (1:100; NB110-55376, Novus Biologicals) and mouse anti- 2,2,7-trimethylguanosine (TMG) antibody (1:50; MABE302, Millipore) for snRNPs, and incubated at 4°C for 24 h. Subsequently, slides were washed with PBS and incubated with secondary antibodies, anti-mouse IgG (whole molecule) F(ab′)2 fragment–Cy3 antibody (1:100, C2181, Sigma-Aldrich) or anti-rabbit IgG (whole molecule)-FITC antibody (1:100, F9887, Sigma-Aldrich) for 1 h at room temperature. After washes in PBS, the sections were mounted with VECTASHIELD (Vector Laboratories), and observed under a Zeiss Axioscope fluorescence microscope (Carl Zeiss), a Nikon C1Si spectral confocal microscope (Nikon Instruments Inc.), or Nikon A1R MP+ multiphoton confocal microscope (Nikon Instruments Inc.).

### Morphometric analysis of DRG sensory neurons

To estimate the neuronal and nuclear size of DRG sensory neurons in 16-week-old diabetic mice and controls, NeuN-stained 10-μm sections from the L4 and L5 DRG were randomly selected for each mouse. The neuronal and nuclear sizes in sensory neurons were measured at a magnification of 400× in sections on a fluorescence microscope (Zeiss Axioscope) coupled to a digital camera with a computer-assisted image analyzer [ImageJ software (NIH)]. The analysis was performed in at least 100 neurons/animal with DAPI- and NeuN-positive nuclei. All analyses were performed with the examiner blinded to the identity of the samples being studied.

### Quantification of CBs, nucleoli and SMN nuclear foci of DRG sensory neurons

CBs are defined by the presence of the marker protein coilin. Nucleoli are enriched in fibrillarin, which is a marker protein for nucleoli; however, CBs also share fibrillarin with nucleoli (Machyna et al., 2013). Therefore, we identified nucleoli as fibrillarin-positive and coilin-negative bodies. The quantitative analysis of number of CBs, nucleoli or SMN nuclear foci was performed in randomly selected 10-μm sections of DRG double-stained, counted in blinded fashion by direct observation of nuclear focal planes at 40× for at least 100 nuclei/animal of DRG sensory neurons (Zeiss Axioscope).

### Quantification of the nuclear TMG staining pattern of DRG sensory neurons

To detect distribution of snRNPs in sensory neurons, anti-TMG antibody, which recognizes the 5′ cap structure of snRNPs, was used. Whether the nucleus of a sensory neuron expressed a multiple dots or a homogeneous nuclear TMG staining pattern was analysed by direct observation throughout the nuclei using a 40× objective for at least 100 nuclei/animal of DRG sensory neurons in blinded fashion.

### RNA isolation and qRT-PCR

Quantitative real-time PCR was performed according to previous descriptions (Christie et al., 2010). Briefly, total RNA was extracted using TRIzol reagent (Invitrogen) as per the manufacturer's instructions. One microgram of total RNA was treated with DNase (Promega) and processed to cDNA synthesis using the Superscript II reverse transcription kit (Invitrogen). All PCR primers were designed using Primer Express 2.0 software (Applied Biosystems). Primer sequences are shown in Table 1. Gene expression analysis by quantification of amplified products was performed using the SYBR Green I fluorophore (Invitrogen). The cycle number at which the fluorescence signal crossed a fixed threshold (threshold cycle, CT) with an exponential growth of PCR product during the linear phase, was recorded. Relative expression values were generated using the comparative CT method (2^−ΔΔCT^) where all genes of interest were normalized to expression of 18S rRNA or RPLPO for mRNA and of 5.8S for small RNA.

**Table 1.**
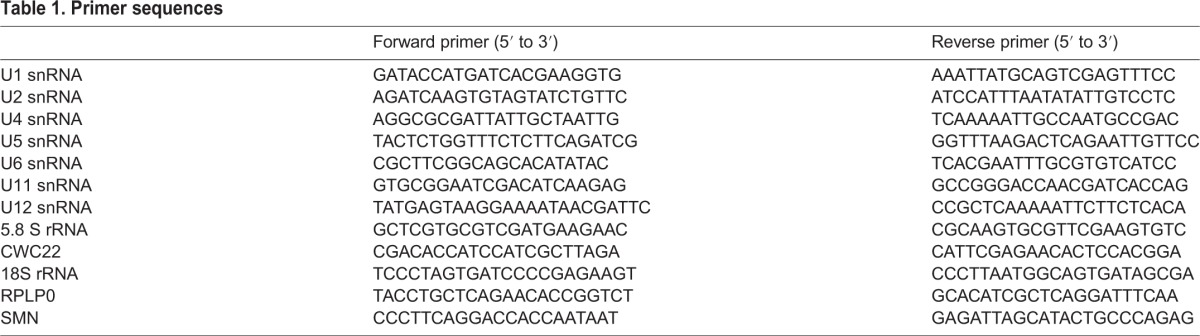
**Primer sequences**

### *In vitro* studies of adult sensory neurons

Dissociated adult sensory neuron culture protocols using rat DRG were prepared as described previously (Christie et al., 2010). Briefly, L4 to L6 DRG were harvested from adult Sprague Dawley rats and placed into L15 medium (Invitrogen). Cells were placed onto poly-L-lysine (Sigma-Aldrich) and 10 µg/ml mouse laminin-coated (Invitrogen) plates. At the time of plating, a 100 nM concentration of CWC22 siRNA (#SI01808695, target sequence 5′-AACATTCTAGAGAATCGAAGA-3′) was added to the culture medium with HiPerfect Transfection Reagent (Qiagen) in the treatment group. The cells were grown for 36 h and then fixed and processed for immunocytochemistry. The sensory neurons and neurites were labelled using primary antibody against mouse NF-200 (1:800; MAB5266, Millipore). The secondary antibody was anti-mouse immunoglobulin G (IgG) CY3 conjugate (1:100; C2181, Sigma-Aldrich) counterstained with DAPI for nuclei. Total neurite outgrowth was analysed and quantified by WIS-Neuromath (Weizmann Institute of Science, Rehovot, Israel) (Galun et al., 2007; Rishal et al., 2012) in blinded fashion. The DRG neurons from the same harvesting were paired with or without CWC22 siRNA treatment.

### Near-nerve injection of CWC22 siRNA *in vivo* studies

Diabetic (3 months of diabetes) and nondiabetic mice underwent near-sciatic–tibial nerve and intraplantar injection of CWC22 siRNA into a left limb, or of scrambled siRNA [1.56 µl or 0.5 µg siRNA with 20 µl HiPerfect transfection reagent (Qiagen) plus 3.44 µl saline (25 µl for footpad) or 28.44 µl saline (50 µl for near nerve)] into a right limb, for 0, 2, 4 and 6 days after injection. Before and 28 days after injection, electrophysiological and behavioural testing as mentioned above were performed. At endpoint (28 days) bilateral L4 to L6 DRGs were harvested for RNA isolation and qRT-PCR.

### Statistical analysis

Results are represented as mean±standard error of the mean (s.e.m.) and compared with Student's *t*-tests (one-tailed in an expected direction of change; two-tailed unless otherwise specified) as appropriate.

## References

[DMM028225C1] Andrade L. E., Chan E. K., Raska I., Peebles C. L., Roos G. and Tan E. M. (1991). Human autoantibody to a novel protein of the nuclear coiled body: immunological characterization and cDNA cloning of p80-coilin. *J. Exp. Med.* 173, 1407-1419. 10.1084/jem.173.6.1407 2033369 PMC2190846

[DMM028225C2] Barbosa I., Haque N., Fiorini F., Barrandon C., Tomasetto C., Blanchette M. and Le Hir H. (2012). Human CWC22 escorts the helicase eIF4AIII to spliceosomes and promotes exon junction complex assembly. *Nat. Struct. Mol. Biol.* 19, 983-990. 10.1038/nsmb.2380 22961380

[DMM028225C3] Berciano M. T., Novell M., Villagra N. T., Casafont I., Bengoechea R., Val-Bernal J. F. and Lafarga M. (2007). Cajal body number and nucleolar size correlate with the cell body mass in human sensory ganglia neurons. *J. Struct. Biol.* 158, 410-420. 10.1016/j.jsb.2006.12.008 17275332

[DMM028225C4] Boulon S., Westman B. J., Hutten S., Boisvert F.-M. and Lamond A. I. (2010). The nucleolus under stress. *Mol. Cell* 40, 216-227. 10.1016/j.molcel.2010.09.024 20965417 PMC2987465

[DMM028225C5] Brown M. J. and Asbury A. K. (1984). Diabetic neuropathy. *Ann. Neurol.* 15, 2-12. 10.1002/ana.410150103 6370098

[DMM028225C6] Brussee V., Cunningham F. A. and Zochodne D. W. (2004). Direct insulin signaling of neurons reverses diabetic neuropathy. *Diabetes* 53, 1824-1830. 10.2337/diabetes.53.7.1824 15220207

[DMM028225C7] Cauchi R. J. (2010). SMN and Gemins: “We are family” … or are we?: Insights into the partnership between Gemins and the spinal muscular atrophy disease protein SMN*. BioEssays* 32, 1077-1089. 10.1002/bies.201000088 20954180

[DMM028225C8] Cheng C. and Zochodne D. W. (2003). Sensory neurons with activated caspase-3 survive long-term experimental diabetes. *Diabetes* 52, 2363-2371. 10.2337/diabetes.52.9.2363 12941777

[DMM028225C9] Cheng C., Kobayashi M., Martinez J. A., Ng H., Moser J. J., Wang X., Singh V., Fritzler M. J. and Zochodne D. W. (2015). Evidence for epigenetic regulation of gene expression and function in chronic experimental diabetic neuropathy. *J. Neuropathol. Exp. Neurol.* 74, 804-817. 10.1097/NEN.0000000000000219 26172287

[DMM028225C10] Christie K. J., Webber C. A., Martinez J. A., Singh B. and Zochodne D. W. (2010). PTEN inhibition to facilitate intrinsic regenerative outgrowth of adult peripheral axons. *J. Neurosci.* 30, 9306-9315. 10.1523/JNEUROSCI.6271-09.2010 20610765 PMC6632469

[DMM028225C11] Christie K. J., Krishnan A., Martinez J. A., Purdy K., Singh B., Eaton S. and Zochodne D. (2014). Enhancing adult nerve regeneration through the knockdown of retinoblastoma protein. *Nat. Commun.* 5, 3670 10.1038/ncomms4670 24752312 PMC5028199

[DMM028225C12] Galun M., Basri R. and Brandt A. (2007). Multiscale edge detection and fiber enhancement using differences of oriented means. *2007 IEEE 11th International Conference on Computer Vision*, pp. 1-8. 10.1109/iccv.2007.4408920

[DMM028225C13] Girard C., Neel H., Bertrand E. and Bordonné R. (2006). Depletion of SMN by RNA interference in HeLa cells induces defects in Cajal body formation. *Nucleic Acids Res.* 34, 2925-2932. 10.1093/nar/gkl374 16738131 PMC1474063

[DMM028225C14] Girard C., Will C. L., Peng J., Makarov E. M., Kastner B., Lemm I., Urlaub H., Hartmuth K. and Lührmann R. (2012). Post-transcriptional spliceosomes are retained in nuclear speckles until splicing completion. *Nat. Commun.* 3, 994 10.1038/ncomms1998 22871813

[DMM028225C15] Grzeschik S. M., Ganta M., Prior T. W., Heavlin W. D. and Wang C. H. (2005). Hydroxyurea enhances SMN2 gene expression in spinal muscular atrophy cells. *Ann. Neurol.* 58, 194-202. 10.1002/ana.20548 16049920

[DMM028225C16] Guo G. F., Kan M., Martinez J. A. and Zochodne D. W. (2011). Local insulin and the rapid regrowth of diabetic epidermal axons. *Neurobiol. Dis.* 43, 414-421. 10.1016/j.nbd.2011.04.012 21530660

[DMM028225C17] Hebert M. D., Szymczyk P. W., Shpargel K. B. and Matera A. G. (2001). Coilin forms the bridge between Cajal bodies and SMN, the Spinal Muscular Atrophy protein. *Genes Dev.* 15, 2720-2729. 10.1101/gad.908401 11641277 PMC312817

[DMM028225C18] Jablonka S., Karle K., Sandner B., Andreassi C., von Au K. and Sendtner M. (2006). Distinct and overlapping alterations in motor and sensory neurons in a mouse model of spinal muscular atrophy. *Hum. Mol. Genet.* 15, 511-518. 10.1093/hmg/ddi467 16396995

[DMM028225C19] Kan M., Guo G., Singh B., Singh V. and Zochodne D. W. (2012). Glucagon-Like Peptide 1, Insulin, Sensory Neurons, and Diabetic Neuropathy. *J. Neuropathol. Exp. Neurol.* 71, 494-510. 10.1097/NEN.0b013e3182580673 22588388

[DMM028225C20] Kariya S., Re D. B., Jacquier A., Nelson K., Przedborski S. and Monani U. R. (2012). Mutant superoxide dismutase 1 (SOD1), a cause of amyotrophic lateral sclerosis, disrupts the recruitment of SMN, the spinal muscular atrophy protein to nuclear cajal bodies. *Hum. Mol. Genet.* 21, 3421-3434. 10.1093/hmg/dds174 22581780 PMC3392116

[DMM028225C21] Kishi M., Tanabe J., Schmelzer J. D. and Low P. A. (2002). Morphometry of dorsal root ganglion in chronic experimental diabetic neuropathy. *Diabetes* 51, 819-824. 10.2337/diabetes.51.3.819 11872686

[DMM028225C22] Klingauf M., Stanĕk D. and Neugebaue K. M. (2006). Enhancement of U4/U6 small nuclear ribonucleoprotein particle association in Cajal bodies predicted by mathematical modeling. *Mol. Biol. Cell* 17, 4972-4981. 10.1091/mbc.E06-06-0513 16987958 PMC1679666

[DMM028225C23] Korngut L., Ma C. H. E., Martinez J. A., Toth C. C., Guo G. F., Singh V., Woolf C. J. and Zochodne D. W. (2012). Overexpression of human HSP27 protects sensory neurons from diabetes. *Neurobiol. Dis.* 47, 436-443. 10.1016/j.nbd.2012.04.017 22569359 PMC3392489

[DMM028225C24] Lefebvre S., Burlet P., Liu Q., Bertrandy S., Clermont O., Munnich A., Dreyfuss G. and Melki J. (1997). Correlation between severity and SMN protein level in spinal muscular atrophy. *Nat. Genet.* 16, 265-269. 10.1038/ng0797-265 9207792

[DMM028225C25] Ling S.-C., Polymenidou M. and Cleveland D. W. (2013). Converging mechanisms in als and FTD: Disrupted RNA and protein homeostasis. *Neuron* 79, 416-438. 10.1016/j.neuron.2013.07.033 23931993 PMC4411085

[DMM028225C26] Liu Q. and Dreyfuss G. (1996). A novel nuclear structure containing the survival of motor neurons protein. *EMBO J.* 15, 3555-3565. 8670859 PMC451956

[DMM028225C27] Lunn M. R. and Wang C. H. (2008). Spinal muscular atrophy. *Lancet* 371, 2120-2133. 10.1016/S0140-6736(08)60921-6 18572081

[DMM028225C28] Machyna M., Heyn P. and Neugebauer K. M. (2013). Cajal bodies: Where form meets function. *Wiley Interdiscip. Rev. RNA* 4, 17-34. 10.1002/wrna.1139 23042601

[DMM028225C29] Mao Y. S., Zhang B. and Spector D. L. (2011). Biogenesis and function of nuclear bodies. *Trends Genet.* 27, 295-306. 10.1016/j.tig.2011.05.006 21680045 PMC3144265

[DMM028225C30] Matera A. G. and Wang Z. (2014). A day in the life of the spliceosome. *Nat. Rev. Mol. Cell Biol.* 15, 108-121. 10.1038/nrm3742 24452469 PMC4060434

[DMM028225C31] Morimoto M. and Boerkoel C. F. (2013). The role of nuclear bodies in gene expression and disease. *Biology (Basel).* 2, 976-1033. 10.3390/biology2030976 24040563 PMC3771687

[DMM028225C32] Narayanan U., Achsel T., Lührmann R. and Matera A. G. (2004). Coupled in vitro import of U snRNPs and SMN, the spinal muscular atrophy protein. *Mol. Cell* 16, 223-234. 10.1016/j.molcel.2004.09.024 15494309

[DMM028225C33] Novotný I., Malinová A., Stejskalová E., Matějů D., Klimešová K., Roithová A., Švéda M., Knejzlík Z. and Staněk D. (2015). SART3-Dependent Accumulation of Incomplete Spliceosomal snRNPs in Cajal Bodies. *Cell Rep.* 10, 429-440. 10.1016/j.celrep.2014.12.030 25600876

[DMM028225C34] Palanca A., Casafont I., Berciano M. T. and Lafarga M. (2014). Reactive nucleolar and Cajal body responses to proteasome inhibition in sensory ganglion neurons. *Biochim. Biophys. Acta* 1842, 848-859. 10.1016/j.bbadis.2013.11.016 24269586

[DMM028225C35] Rishal I., Golani O., Rajman M., Costa B., Ben-Yaakov K., Schoenmann Z., Yaron A., Basri R., Fainzilber M. and Galun M. (2012). WIS-NeuroMath enables versatile high throughput analyses of neuronal processes. *Dev. Neurobiol*. 2013;73:247-256. 10.1002/dneu.22061 23055261

[DMM028225C36] Rudnik-Schöneborn S., Goebel H. H., Schlote W., Molaian S. and Omran H. (2003). Classical infantile spinal muscular atrophy with SMN deficiency causes sensory neuronopathy. *Neurology* 60, 983-987. 10.1212/01.WNL.0000052788.39340.45 12654964

[DMM028225C37] Singh B., Singh V., Krishnan A., Koshy K., Martinez J. A., Cheng C., Almquist C. and Zochodne D. W. (2014). Regeneration of diabetic axons is enhanced by selective knockdown of the PTEN gene. *Brain* 137, 1051-1067. 10.1093/brain/awu031 24578546 PMC3959560

[DMM028225C38] Singhal A., Cheng C., Sun H. and Zochodne D. W. (1997). Near nerve local insulin prevents conduction slowing in experimental diabetes. *Brain Res.* 763, 209-214. 10.1016/S0006-8993(97)00412-5 9296561

[DMM028225C39] Steckelberg A.-L., Boehm V., Gromadzka A. M. and Gehring N. H. (2012). CWC22 connects pre-mRNA splicing and exon junction complex assembly. *Cell Rep.* 2, 454-461. 10.1016/j.celrep.2012.08.017 22959432

[DMM028225C40] Steckelberg A.-L., Altmueller J., Dieterich C. and Gehring N. H. (2015). CWC22-dependent pre-mRNA splicing and eIF4A3 binding enables global deposition of exon junction complexes. *Nucleic Acids Res.* 43, 4687-4700. 10.1093/nar/gkv320 25870412 PMC4482076

[DMM028225C41] Strzelecka M., Oates A. C. and Neugebauer K. M. (2010a). Dynamic control of Cajal body number during zebrafish embryogenesis. *Nucleus* 1, 96-108. 10.4161/nucl.1.1.10680 21327108 PMC3035118

[DMM028225C42] Strzelecka M., Trowitzsch S., Weber G., Lührmann R., Oates A. C. and Neugebauer K. M. (2010b). Coilin-dependent snRNP assembly is essential for zebrafish embryogenesis. *Nat. Struct. Mol. Biol.* 17, 403-409. 10.1038/nsmb.1783 20357773

[DMM028225C43] Tsuiji H., Iguchi Y., Furuya A., Kataoka A., Hatsuta H., Atsuta N., Tanaka F., Hashizume Y., Akatsu H., Murayama S. et al. (2013). Spliceosome integrity is defective in the motor neuron diseases ALS and SMA. *EMBO Mol. Med.* 5, 221-234. 10.1002/emmm.201202303 23255347 PMC3569639

[DMM028225C44] Xu Q.-G., Li X.-Q., Kotecha S. A., Cheng C., Sun H. S. and Zochodne D. W. (2004). Insulin as an in vivo growth factor. *Exp. Neurol.* 188, 43-51. 10.1016/j.expneurol.2004.03.008 15191801

[DMM028225C45] Young P. J., Le T. T., thi Man N., Burghes A. H. M. and Morris G. E. (2000). The relationship between SMN, the spinal muscular atrophy protein, and nuclear coiled bodies in differentiated tissues and cultured cells. *Exp. Cell Res.* 256, 365-374. 10.1006/excr.2000.4858 10772809

[DMM028225C46] Zhang Z., Lotti F., Dittmar K., Younis I., Wan L., Kasim M. and Dreyfuss G. (2008). SMN deficiency causes tissue-specific perturbations in the repertoire of snRNAs and widespread defects in splicing. *Cell* 133, 585-600. 10.1016/j.cell.2008.03.031 18485868 PMC2446403

[DMM028225C47] Zochodne D. W. (2007). Diabetes mellitus and the peripheral nervous system: manifestations and mechanisms. *Muscle Nerve* 36, 144-166. 10.1002/mus.20785 17469109

[DMM028225C48] Zochodne D. W. (2015). Diabetes and the plasticity of sensory neurons. *Neurosci. Lett.* 596, 60-65. 10.1016/j.neulet.2014.11.017 25445357

